# Mucosal Advancement Flap Versus Ligation of the Intersphincteric Fistula Tract for Transsphincteric Fistula-in-Ano: A Comparative Study in a Tertiary Care Hospital

**DOI:** 10.7759/cureus.99295

**Published:** 2025-12-15

**Authors:** Sagar Reddy G, Ashok Reddy R

**Affiliations:** 1 General Surgery, Great Eastern Medical School and Hospital, Srikakulam, IND

**Keywords:** complex anal fistula, ligation of intersphincteric fistula tract, mucosal advancement flap, recurrent anal fistula, transphincteric anal fistula

## Abstract

Background and aim

Transsphincteric fistula-in-ano (TPAF) is a common anorectal condition that poses significant surgical challenges due to the involvement of the external anal sphincter and the risk of postoperative incontinence. Sphincter-preserving procedures such as the mucosal advancement flap (MAF) and ligation of the intersphincteric fistula tract (LIFT) have become preferred alternatives; however, their comparative outcomes remain debated. This study aimed to compare postoperative outcomes between the MAF and LIFT procedures in patients with TPAF.

Methods

A prospective comparative study was conducted in the Department of General Surgery, Great Eastern Medical School and Hospital, Srikakulam, Andhra Pradesh, from June 2025 to September 2025. A total of 14 patients diagnosed with TPAF were randomized into two groups: Group A (MAF, n = 7) and Group B (LIFT, n = 7). Postoperative pain (Visual Analogue Scale (VAS) score), wound infection, time to complete healing, anal continence (Wexner score), and recurrence were assessed during a one-month follow-up.

Results

The mean age of the study population was 46.8 years, with a male-to-female ratio of 3:2. Postoperative pain at 48 hours was significantly lower in the LIFT group (VAS 3.1 ± 0.6) compared to the MAF group (VAS 4.8 ± 0.9, p = 0.02). Complete wound healing occurred faster with MAF (mean 18.6 ± 3.2 days) than with LIFT (21.4 ± 3.9 days, p = 0.05). Minor wound infection occurred in one patient (14.3%) from each group. No major incontinence was reported in either group, and no recurrence was observed at one month.

Conclusions

Both MAF and LIFT are safe and effective sphincter-preserving techniques for managing TPAF. LIFT offers reduced postoperative pain and shorter operative time, while MAF offers faster healing. Larger, multicenter trials with extended follow-up are warranted to establish long-term efficacy.

## Introduction

A fistula-in-ano is an abnormal connection between the anal canal or rectum and the perianal skin [1]. It primarily affects young adults, with an incidence ranging from 0.86 to 2 cases per 10,000 individuals annually. The condition is more common among smokers, individuals with diabetes, and those with a high BMI. It typically develops following an anorectal abscess and presents with discharge, itching, and pain. Fistula-in-ano causes significant morbidity, imposes a financial burden, and adversely affects quality of life [2-4].

According to Parks’ classification, fistula-in-ano may be intersphincteric, transsphincteric, suprasphincteric, or extrasphincteric [5]. It can also be broadly categorized into low fistulas (subcutaneous, intersphincteric, or low transsphincteric) and high fistulas (high transsphincteric, suprasphincteric, or extrasphincteric) [6]. The American Gastroenterological Association defines transsphincteric fistula-in-ano (TPAF) as a complex fistula [7].

Managing transsphincteric fistulas remains challenging. Effective treatment requires precise identification of the tract and internal opening, complete eradication of the fistula tract, preservation of anal sphincter function, and adequate drainage. Despite advancements, recurrence and incontinence continue to pose major concerns [8-16]. Sphincter-sparing procedures such as the mucosal advancement flap (MAF) and ligation of the intersphincteric fistula tract (LIFT) have gained prominence.

Risk factors for recurrence include complex anatomy, diabetes mellitus, obesity (BMI > 25 kg/m²), inadequate preoperative assessment, and surgical inexperience [4]. Risk factors for postoperative incontinence include complex disease and prior fistula surgery [10,13].

In this study, we hypothesized that LIFT would result in better healing outcomes, reduced postoperative pain, and faster recovery compared to the MAF technique.

## Materials and methods

Study design

This was a prospective comparative study conducted to evaluate and compare postoperative outcomes between MAF and LIFT procedures in patients diagnosed with TPAF.

Duration

The study was conducted in the Department of General Surgery, Great Eastern Medical School and Hospital (GEMS), Srikakulam, Andhra Pradesh, India, from June 2025 to September 2025.

Ethical approval

The study protocol was reviewed and approved by the Institutional Ethics Committee of GEMS & Hospital (approval 107/IEC/GEMS&H/2025). Written informed consent was obtained from all participants after explaining the nature, purpose, and potential risks of the study. The study was conducted in accordance with the principles of the Declaration of Helsinki (2013 revision).

Sample size justification

A total of 14 patients were included, divided equally into two groups. The sample size was determined based on expected patient inflow during the study period and feasibility constraints. A power analysis (α = 0.05, β = 0.20) assuming a 30% difference in complication or recurrence rates between groups indicated a minimum of 12 patients for 80% statistical power. To account for possible loss to follow-up, the total sample size was increased to 14 patients.

Randomization

Patients were randomized into two groups using block randomization (block size of 4) to ensure equal allocation. A computer-generated randomization sequence was prepared by an independent assistant, and allocation concealment was maintained using sealed, opaque, sequentially numbered envelopes opened immediately before surgery by a nurse not involved in outcome assessment. Group A included patients undergoing MAF, while Group B included patients undergoing LIFT.

Patient selection

Patients attending the General Surgery Outpatient Department and diagnosed with TPAF, confirmed clinically and radiologically, were evaluated for eligibility. Inclusion criteria included age above 18 years and clinically and radiologically confirmed TPAF. Exclusion criteria included age below 18 years, other fistula types (intersphincteric, suprasphincteric, and extrasphincteric), associated Crohn’s disease, tuberculosis, or anal malignancy, and patients unfit for anesthesia or those refusing consent.

Preoperative evaluation

All patients underwent a detailed clinical evaluation, including history, local examination, and necessary laboratory investigations. Imaging with MRI of the pelvis or endoanal ultrasonography was performed when required to confirm tract anatomy and the internal opening.

Surgical techniques

All procedures were performed by the same surgeon with over 20 years of experience in colorectal surgery, and standard aseptic precautions were maintained throughout.

MAF Technique

MAF procedures were performed under spinal anesthesia with the patient in the lithotomy position. The internal opening was identified using a fistula probe or by instilling hydrogen peroxide through the external opening. A mucosal-submucosal advancement flap was marked approximately 2-3 cm proximal to the dentate line, with a typical width of 1.5-2 cm, designed to allow tension-free advancement while maintaining adequate vascularity. The flap was elevated using sharp dissection, incorporating mucosa and submucosa, with optional inclusion of a thin layer of circular muscle to enhance blood supply. Care was taken to preserve the vascular pedicle and minimize trauma. The internal opening and adjacent fibrotic tissue were curetted or excised, then closed primarily using 3-0 polyglactin absorbable sutures in an interrupted or layered manner, depending on tissue integrity. The mobilized flap was advanced distally to cover the internal opening without tension, and the edges were sutured to the anoderm or rectal mucosa using absorbable interrupted sutures to ensure a watertight seal. The external opening and remaining fistulous tract were thoroughly curetted to remove granulation tissue, and external wounds were left open to allow drainage and heal by secondary intention. Postoperatively, patients were advised to begin sitz baths twice daily starting on postoperative day 2, along with routine stool softeners, a high-fiber diet, analgesics, and a short course of broad-spectrum antibiotics according to institutional protocol.

LIFT Technique

LIFT procedures were performed under spinal or general anesthesia with the patient in the prone jackknife position. The fistula tract and internal opening were identified using a probe or hydrogen peroxide injection. A 2-3 cm curvilinear incision was made directly over the intersphincteric groove corresponding to the fistula tract. Careful blunt and sharp dissection exposed the intersphincteric portion of the tract, with caution taken to avoid injury to the internal or external sphincters. The fistula tract was isolated, hooked, and looped using a right-angle clamp for stabilization, then ligated proximally near the internal opening and distally using 2-0 absorbable polyglactin sutures, ensuring secure knot tying to prevent slippage or leakage. The segment between the two ligatures was transected, disconnecting the internal opening from the external tract. The remaining distal tract was curetted to remove granulation tissue, while the external opening was left intact unless clinical indications warranted excision. The intersphincteric incision was closed with absorbable interrupted sutures, maintaining anatomical alignment and preventing dead space formation. Postoperative care mirrored that of the MAF group, including twice-daily sitz baths starting on day 2, stool softeners, a high-fiber diet, analgesics, and a short course of broad-spectrum antibiotics.

Outcome parameters

Postoperative outcomes were assessed using standardized and validated clinical measures to ensure uniform comparison between the two surgical groups. Pain intensity at 48 hours was evaluated using the Visual Analogue Scale (VAS), which ranges from 0, indicating no pain, to 10, representing the worst imaginable pain. Wound infection was identified based on the presence of clinical features such as erythema, purulent discharge, or localized warmth, and was further confirmed by microbial culture when indicated. Functional recovery was measured by recording the number of days each patient took to resume routine daily activities, providing an indirect assessment of postoperative discomfort and overall recovery. Anal continence was assessed at one month using the Wexner Continence Score, a validated scoring system ranging from 0 (normal continence) to 20 (complete incontinence), with evaluations performed by an independent assessor to minimize bias. Recurrence was defined as the persistence or reappearance of the fistulous tract or associated discharge from the same site within one month after surgery, determined through clinical examination and supplemented with imaging when necessary.

Follow-up protocol

Patients were followed up at one week, two weeks, and one month postoperatively for wound assessment, continence evaluation, and recurrence monitoring. Compliance and symptom improvement were recorded at each visit.

Statistical analysis

Data were analyzed using IBM SPSS Statistics for Windows, Version 25.0 (Released 2017; IBM Corp., Armonk, NY, USA). Quantitative variables were expressed as mean ± SD and compared using the Student’s t-test, while categorical variables were analyzed using the chi-square test. A p-value of < 0.05 was considered statistically significant.

## Results

**Figure 1:**
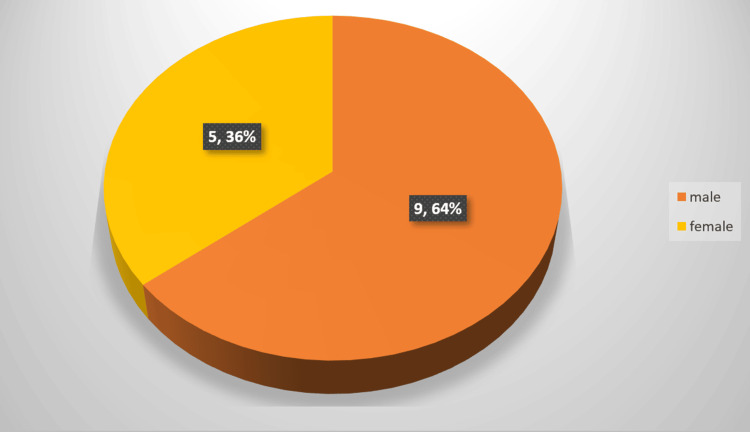
Demographics The orange segment represents male patients (9, 64%), while the yellow segment represents female patients (5, 36%).

A total of 14 patients diagnosed with TPAF were included in the study. The mean age of the study population was 46.8 ± 9.4 years, with a male-to-female ratio of 3:2, as shown in Figure 1.

Demographic distribution and comorbidities

**Figure 2:**
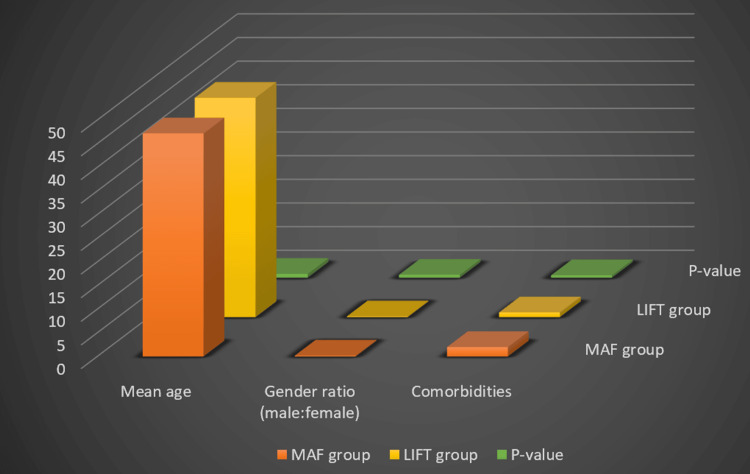
Demographic characteristics This bar chart visually compares baseline characteristics between the MAF and LIFT patient groups. Orange bars represent the MAF group, while yellow bars depict the LIFT group for mean age, gender ratio (male:female), and comorbidities (diabetes/hypertension). Green bars indicate p-values for each parameter, showing no significant differences between the groups across all measured baseline characteristics. LIFT, ligation of the intersphincteric fistula tract; MAF, mucosal advancement flap

Table 1 summarizes the demographic characteristics, including mean age, gender, and comorbidities (diabetes or hypertension). Statistical analysis showed no significant differences between the two groups, indicating that the demographic and clinical profiles were comparable at baseline. This distribution is also illustrated in Figure 2.

**Table 1:** Demographic characteristics This table presents the baseline demographic and clinical characteristics of patients in the MAF and LIFT groups. LIFT, ligation of the intersphincteric fistula tract; MAF, mucosal advancement flap

Parameter	MAF group (n = 7)	LIFT group (n = 7)	p-Value
Mean age (years)	47.2 ± 9.1	46.4 ± 9.8	0.81
Gender (M:F)	5:02	4:03	0.62
Comorbidities (diabetes/hypertension)	2 (28.6%)	1 (14.3%)	0.52

Postoperative pain

Postoperative pain was assessed using the VAS at 48 hours. The mean VAS score was significantly lower in the LIFT group (3.1 ± 0.6; 95% CI: 2.6-3.6) compared to the MAF group (4.8 ± 0.9; 95% CI: 4.1-5.5), demonstrating a statistically significant difference (p = 0.02) as shown in Table 2.

**Table 2:** Comparison of postoperative pain scores (VAS at 48 hours) A statistically significant difference (p < 0.05) indicates lower postoperative pain in the LIFT group. LIFT, ligation of the intersphincteric fistula tract; MAF, mucosal advancement flap; VAS, Visual Analogue Scale

Group	Mean VAS score (48 hours)	SD	95% CI	p-Value
MAF	4.8	0.9	4.1-5.5	0.02
LIFT	3.1	0.6	2.6-3.6	

Wound healing

The mean wound healing time was significantly shorter in the MAF group (18.6 ± 3.2 days; 95% CI: 16.5-20.7) compared to the LIFT group (21.4 ± 3.9 days; 95% CI: 19.0-23.8) (p = 0.05), indicating faster complete wound healing with MAF, as shown in Table 3.

**Table 3:** Comparison of wound healing time Shorter mean healing time in the MAF group indicates faster wound recovery compared to LIFT (p = 0.05). LIFT, ligation of the intersphincteric fistula tract; MAF, mucosal advancement flap

Group	Mean healing time (days)	SD	95% CI	p-Value
MAF	18.6	3.2	16.5 - 20.7	0.05
LIFT	21.4	3.9	19.0 - 23.8	

Complications

Minor surgical site infection occurred in one patient (14.3%) in each group and was managed conservatively with antibiotics. No cases of postoperative bleeding, urinary retention, or wound dehiscence were observed, as shown in Table 4.

**Table 4:** Postoperative complications This table summarizes postoperative complications following MAF and LIFT surgeries. The rate of surgical site infection was identical in both groups (1, 14.3%), and no patients experienced bleeding, urinary retention, or wound dehiscence. A p-value of 1.00 indicates no statistical difference in complication rates, supporting the comparable safety profiles of both procedures. LIFT, ligation of the intersphincteric fistula tract; MAF, mucosal advancement flap

Complication	MAF group (n = 7)	LIFT group (n = 7)	p-Value
Surgical site infection	1 (14.3%)	1 (14.3%)	1.00
Bleeding	0	0	-
Urinary retention	0	0	-
Wound dehiscence	0	0	-

Continence

All patients maintained continence at follow-up. The mean postoperative Wexner continence score at one month was 0.3 ± 0.5 in both groups, as shown in Table 5.

**Table 5:** Postoperative continence outcomes Postoperative fecal continence was evaluated using the Wexner score in patients undergoing MAF and LIFT. The Wexner score ranges from 0 (perfect continence) to 20 (complete incontinence). The low scores in both groups indicate minimal to no incontinence, with no statistically significant difference (p = 1.00), confirming excellent functional outcomes for both procedures. LIFT, ligation of the intersphincteric fistula tract; MAF, mucosal advancement flap

Parameter	MAF group (n = 7)	LIFT group (n = 7)	p-Value
Mean Wexner score	0.3 ± 0.5	0.3 ± 0.5	1.00
Incontinence present	0	0	

Recurrence

All patients completed a one-month follow-up, and no recurrence of the fistula tract was observed in either group.

## Discussion

The findings of the present study demonstrate that both MAF and LIFT are effective sphincter-preserving procedures, with no significant differences in recurrence or continence at one month postoperatively. These results align broadly with the literature. Qureshi et al. (2018) reported similar healing rates between MAF and LIFT in elderly patients, though MAF was associated with better continence, highlighting a trade-off between functional outcomes and perioperative risks [1]. This underscores the importance of individualized surgical decision-making based on patient priorities.

A meta-analysis by Stellingwerf et al. (2019) further supports comparable success and recurrence rates between endorectal advancement flap (a technique akin to MAF) and LIFT, with significantly better continence preservation following LIFT [3]. This corresponds with the lower postoperative pain observed with LIFT in the present study, reflecting its minimally invasive nature and reduced disruption of sphincter integrity.

Zahra et al. (2022) systematically reviewed various surgical techniques, including MAF and LIFT, but noted considerable variability in surgical methods, inclusion criteria, and follow-up, limiting definitive comparisons [2]. They emphasized that no single technique universally outperforms others in complex anal fistula management, advocating for larger, standardized randomized controlled trials (RCTs) to provide robust comparative data.

The results of this study are also consistent with the observations of Mohanlal et al. (2016), who evaluated outcomes of various surgical approaches for fistula-in-ano, with particular focus on LIFT [6]. They reported that LIFT is a reliable sphincter-preserving option with encouraging healing rates and minimal risk of postoperative incontinence, particularly in transsphincteric fistulas. Their study highlighted LIFT as a simple, safe procedure with low morbidity, an advantage mirrored in our results, where patients undergoing LIFT demonstrated lower postoperative pain scores and maintained excellent continence at one-month follow-up. Mohanlal et al. also emphasized that careful identification of the intersphincteric plane and secure ligation of the fistula tract are critical for optimizing outcomes [6]. The alignment between our findings and theirs further supports LIFT as an effective, patient-friendly alternative to more invasive flap procedures, especially where early postoperative recovery and preservation of sphincter function are priorities.

Xu et al. (2024) reported variable healing rates with advancement flap repairs but noted that combining these techniques with LIFT might improve outcomes, suggesting that evolving hybrid approaches could optimize the benefits of both procedures [9]. Overall, recent studies collectively indicate that MAF and LIFT provide comparable healing outcomes for complex transsphincteric fistulas but differ in early postoperative recovery and functional implications such as continence preservation [17-20]. Surgical technique selection should therefore be patient-centered, incorporating fistula complexity, sphincter function, and patient preferences to balance short-term recovery with long-term functional outcomes.

Longer-term, multicenter randomized studies with standardized methodologies and extended follow-up are necessary to establish evidence-based surgical guidelines for the optimal management of anal fistulas.

Limitations

This study is limited by its small sample size and short follow-up duration, which restricts the generalizability of the findings. Resource constraints and the short study period limited the feasibility of recruiting a larger cohort. Additionally, the study was conducted in a single tertiary care center. Despite these limitations, the study provides preliminary comparative data on two sphincter-preserving techniques for TPAF, forming a foundation for larger, multicentric trials with extended follow-up.

## Conclusions

Both MAF and LIFT are sphincter-preserving procedures for managing TPAF. LIFT offers the advantage of reduced postoperative pain, providing a more comfortable recovery for patients, while MAF remains an excellent option with faster wound healing. Technical refinements in both MAF and LIFT, including aggressive closure of the internal opening, use of well-vascularized flaps, standardized postoperative care, and careful wound management, have contributed to improved healing rates and reduced recurrence. Hybrid approaches and adjuncts, such as laser coagulation, further highlight the importance of personalized, multimodal treatment plans that leverage the strengths of multiple techniques.

Future multicenter RCTs using standardized surgical protocols and extended follow-up are essential to establish robust, evidence-based guidelines for the optimal management of complex anal fistulas. Integrating patient-reported outcomes and multidisciplinary perspectives will further enhance the precision and quality of surgical care in this challenging condition.
